# Vancomycin wound penetration in open-heart surgery patients receiving negative pressure wound therapy for deep sternal wound infection

**DOI:** 10.1080/07853890.2024.2444544

**Published:** 2024-12-23

**Authors:** Martin Kolek, Jana Ďuricová, Hana Brozmanová, Pavel Šištík, Jan Juřica, Klára Kaňková, Oldřich Motyka, Ivana Kacířová

**Affiliations:** a Department of Cardiac Surgery, University Hospital Ostrava, Ostrava, Czech Republic; b Department of Clinic Subjects, Faculty of Medicine, University of Ostrava, Ostrava, Czech Republic; c Department of Clinical Pharmacology, Institute of Laboratory Medicine, University Hospital Ostrava, Ostrava, Czech Republic; d Department of Clinical Pharmacology, Faculty of Medicine, University of Ostrava, Ostrava, Czech Republic; e Department of Pharmacology, Faculty of Medicine, Masaryk University, Brno, Czech Republic; f Hospital Pharmacy, Masaryk Memorial Cancer Institute, Brno, Czech Republic; g Department of Environmental Engineering, Faculty of Mining and Geology, VŠB - Technical University of Ostrava, Ostrava, Czech Republic

**Keywords:** Deep sternal wound infection, exudate, negative pressure wound therapy, open-heart surgery, vancomycin, wound penetration

## Abstract

**Introduction:**

It is hypothesized that systemically administered antibiotics penetrate wound sites more effectively during negative pressure wound therapy (NPWT). However, there is a lack of clinical data from patients who receive NPWT for deep sternal wound infection (DSWI) after open-heart surgery. Here, we evaluated vancomycin penetration into exudate in this patient group.

**Patients and methods:**

For this prospective observational study, we enrolled 10 consecutive patients treated with NPWT for post-sternotomy DSWI. On the first sampling day, serum and exudate samples were synchronously collected at 0 (pre-dose), 0.5, 1, 2, 3 and 6 h after vancomycin administration. On the following three consecutive days, additional samples were collected, only before vancomycin administration.

**Results:**

The ratio of average vancomycin concentration in wound exudate to in serum was higher for free (unbound) (1.51 ± 0.53) than for total (bound + unbound) (0.91 ± 0.29) concentration (*p* = 0.049). The percentage of free vancomycin was higher in wound exudate than serum (0.79 ± 0.19 vs. 0.46 ± 0.16; *p* = 0.04). Good vancomycin wound penetration was maintained on the following three days (vancomycin trough exudate-to-serum concentration ratio > 1). The total hospital stay was significantly longer in patients with DSWI (46 ± 11.6 days) versus without DSWI (14 ± 11.7 days) (*p* < 0.001). There was no in-hospital or 90-day mortality. Two patients experienced late DSWI recurrence. All-cause mortality was 4.8% during a median follow-up of 2.5 years.

**Conclusion:**

Vancomycin effectively penetrates wound exudate in patients receiving NPWT for DSWI after open-heart surgery.

The protocol for this study was registered at ClinicalTrials.gov on July 16, 2024 (NCT06506032).

## KEY MESSAGES

- Vancomycin effectively penetrates into wound exudate in patients receiving NPWT to treat post-sternotomy DSWI after open-heart surgery.
- NPWT promotes the penetration of vancomycin into the infection site.

## Introduction

After open-heart surgery, one of the most serious complications is deep sternal wound infection (DSWI), which is associated with significantly increased in-hospital mortality (up to 14%) and decreased long-term survival [1–5]. Reported DSWI incidence rates range from 0.2–3% [1, 3, 4, 6–9]. Deep infection extends below the fascia and can involve the bone (sternum osteomyelitis) or retrosternal tissue (mediastinitis). The most common causative pathogens are coagulase-negative staphylococci, followed by *Staphylococcus aureus*, including methicillin-resistant *Staphylococcus aureus* (MRSA) [3, 10, 11].

DSWI management includes antibiotic therapy, surgical removal of necrotic tissues, negative pressure wound therapy (NPWT) and subsequent secondary sternal closure. Successful treatment of the infection requires achieving an adequate antibiotic concentration in the tissues of the infected wound. In general, it has been believed that most antibiotics reach equilibrium in tissues and plasma; however, recent studies show high inter-tissue and inter-subject variability in antibiotic distribution, and substantial differences in drug concentrations at the target site versus in plasma [12, 13].

Application of local negative pressure is currently the primary treatment strategy for DSWI. It is hypothesized that systemically administered antibiotics more effectively penetrate to the wound site during NPWT. A higher antibiotic concentration in the infected tissue should yield a more intense bactericidal effect. Few published studies have monitored the penetration of antibiotics from blood into exudate in patients treated with NPWT and have evaluated the adequacy of current dosage regimens based on antibiotic tissue concentrations [12, 14, 15]. To our knowledge, no similar study has been published in the field of cardiac surgery, and there are currently no guidelines regarding the target exudate/tissue concentration of vancomycin. Thus, in the present study, we evaluated the pharmacokinetic profile and wound penetration of vancomycin in open-heart surgery patients receiving NPWT for post-sternotomy DSWI.

## Patients and methods

### Ethics

The study was approved by the Ethics Committee of University Hospital Ostrava, Czech Republic (approval number 332/2021, 29 April 2021, protocol code number 06/RVO-FNOs/2021). All included patients received written information about the study, gave their written informed consent prior to enrolment and agreed to the publication of the results. This study adheres to the policies and requirements described in the World Medical Association Declaration of Helsinki. The protocol for this study was registered at ClinicalTrials.gov on July 16, 2024 (NCT06506032).

### Patients

For this prospective observational clinical study, we enrolled 10 consecutive patients who were treated with NPWT for DSWI after open-heart surgery, between July 2021 and November 2023. Patients were eligible if they met the following inclusion criteria: age ≥ 18 years, clinical and laboratory signs of significant infection, and an indication for NPWT with concomitant antibiotic therapy.

### DSWI diagnosis

Following the criteria of the Centers for Disease Control and Prevention, DSWI was defined by the presence of at least one of the following findings: (1) identification of organisms in mediastinal tissue or fluid by a culture-based or nonculture-based microbiologic testing method; (2) evidence of mediastinitis observed intraoperatively or by histopathologic examination; and (3) at least one of the following signs or symptoms – fever (>38.0 °C), chest pain, or sternal instability – as well as purulent drainage from the mediastinum or mediastinal widening on imaging [16].

### Study design and sampling schedule

NPWT was initiated within 24 h of vancomycin introduction. It was administered using a Vivano^®^Tec Pro Unit (Paul Hartmann AG, Germany), with continuous negative pressure of −125 mmHg. Wound dressings were changed three times weekly under aseptic conditions, under general anaesthesia. At predefined time intervals after antibiotic administration, wound exudate was collected from the tube connecting the wound dressing set and canister, to determine the antibiotic concentration. NPWT was discontinued when the following criteria were met: absence of fever, wound was macroscopically infection-free, decreased laboratory inflammatory markers (C-reactive protein and leukocyte count), and negative wound culture.

Vancomycin solution was intravenously administered over a period of 0.5–2.0 h. The initial dose was determined according to the patient’s body weight: 15–20 mg/kg body weight, every 8–12 h. During the course of therapy, the vancomycin dose was adjusted according to therapeutic drug monitoring (TDM) rules, to achieve the desired target serum concentration. The aim was to achieve a vancomycin serum trough concentration (i.e. before administration) in the range of 10–20 mg/l [17]. In patients at increased risk of vancomycin-induced nephrotoxicity, vancomycin concentrations and renal function were monitored more frequently during treatment (at the individual discretion of the attending physician).

On the first sampling day, serum and exudate samples were synchronously collected at 0 (pre-dose), 0.5, 1, 2, 3 and 6 h after vancomycin administration. On the following three consecutive days, additional collections were performed, only before administration. Blood samples were immediately centrifuged, and serum was aliquoted into separated tubes. Serum and exudate samples were frozen at −80 °C until analysis.

### Sample preparation and high-performance liquid chromatography (HPLC) analysis

Vancomycin in serum and exudate was analysed using our previously developed method. Briefly, for both matrices, 50 µl was precipitated with 20 µl of 33% trichloroacetic acid and 200 µl of deionized water. This mixture was vortexed, and then 50 µl of acetonitrile and 50 µl of 0.5 mol/l NH_4_OH were added. This mixture was centrifuged, and then 10 µl from the upper layer was injected into the chromatographic system [18]. We processed 500 µl aliquots of serum or exudate using the Microcon^®^ Centrifugal Filter Unit, to obtain ultrafiltrate. To quantify the concentration of free (unbound) vancomycin, we analysed 50 µl of ultrafiltrate [19]. Vancomycin was analysed using a UHPLC-MS/MS system [18].

### Pharmacokinetic calculations and statistical analysis

We compared the vancomycin concentrations between exudate from NPWT sites versus in serum. To determine the vancomycin penetration into the wound during NPWT, we compared the vancomycin total (bound + unbound) average exudate concentration to the vancomycin total (bound + unbound) average serum concentration on the first sampling day. The same calculation was used to determine the penetration ratio for free (unbound) vancomycin concentrations on the first sampling day. For samples from the following three consecutive days, we compared the vancomycin total (bound + unbound) trough exudate concentrations with the vancomycin total (bound + unbound) trough serum concentrations. The same calculation was used to determine the penetration ratio for free (unbound) vancomycin concentrations on the three consecutive days. To calculate the free (unbound) vancomycin ratio in serum and exudate, we divided the free (unbound) average vancomycin concentration by the total (bound + unbound) average vancomycin concentration.

Pharmacokinetic parameters were calculated using Kinetica 4.4.1 pharmacokinetic software (Thermo Electron, Waltham, MA, USA). The area under the concentration–time curve (AUC_0–t_) was calculated using the linear trapezoidal rule, from time zero to the last sampling, exceeding the limit of quantification (LOQ). The average serum and exudate concentrations were calculated as the area under the curve divided by 24 h (C_average_ = AUC_0–24_/24). The AUC from time zero to 24 h (AUC_0–24_) was calculated as AUC_t–dosing int_ * the number of dosing intervals per 24 h. The AUC_t–dosing int_ was the extrapolated portion of the AUC from the last sampling to the time of the end of the dosing interval in each patient (i.e. 8–12 h, according to the patient’s dosing schedule). This calculation was based on the linear regression of the logarithmic transformation of the last data points of the curve.

All statistical analyses and visualisations were performed in the R environment (R core group, 2024, R Foundation for Statistical Computing, Vienna, Austria) [20]. A significance level of 0.05 was used for all statistical tests. Two-sample *t*-tests were used to compare two quantitative variables, and one-sample *t*-tests were used to compare one quantitative variable to a constant. Fisher’s exact test was used for the assessment of qualitative variables and percentages, since the number of observations in one category was always ≤5.

## Results

### Patients’ clinical characteristics

The DSWI incidence observed during the study period was 1.8% (21 of 1190 operated patients). Table 1 depicts the clinical characteristics of the 10 study subjects treated with vancomycin. The male-to-female ratio was 7:3, mean age was 66 ± 10 years, and mean weight was 101.1 ± 14.4 kg. The majority of patients (7 of 10) had undergone coronary artery bypass graft (CABG) surgery, performed alone or in combination with valve surgery. DSWI was classified based on the involved anatomic layer [21, 22], and 80% of patients had type 2 C DSWI (bone and retrosternal tissue involvement), and 20% had type 2 A DSWI (without bone or retrosternal tissue involvement). At the time of starting negative pressure and antibiotic therapy, the mean C-reactive protein concentration was 159 ± 112 mg/l. On the first sampling day, the mean daily vancomycin dosage was 2200 ± 400 mg. The first parallel serum and exudate sampling was predominantly performed on day 3 of vancomycin treatment (range: days 2–6).

**Table 1.** Clinical characteristics of patients.

Patient No.	Sex	Age, years	Weight, kg	Height, cm	BMI, kg/m^2^	Primary heart surgery	Type of DSWI based on anatomical layer involved	Initial C-reactive protein, mg/l	Daily vancomycin dose on 1^st^ sampling day, mg	Time to 1^st^ sample collection, days
1	F	77	98.0	165	36.7	supracoronary ascending aortic and hemiarch replacement	2A	133	2000	3
2	M	59	93.8	167	33.7	CABG + MV repair + TV repair	2C	217	2000	3
3	F	81	106.0	162	40.4	AV replacement + MV replacement	2C	155	2000	5
4	M	64	69.1	166	25.1	CABG	2C	276	2000	3
5	M	53	128.0	173	42.8	CABG	2C	324	2000	3
6	M	71	105.0	174	34.7	CABG + MV repair	2C	46	2000	3
7	M	57	94.0	174	31.1	CABG	2A	13	2000	3
8	M	53	112.0	174	37.0	CABG	2C	5	3000	3
9	M	75	97.5	170	33.7	CABG	2C	260	3000	2
10	F	73	108.0	164	38.0	MV replacement + TV repair	2C	164	2000	6
Mean ± SD		66 ± 10	101.1 ± 14.4	168.9 ± 4.4	35.3 ± 4.7			159 ± 112	2200 ± 400	3.4 ± 1.1
Median		68	101.5	169	35.7			160	2000	3
Range		53–81	69.1–128.0	162–174	25.1–42.8			5–324	2000–3000	2–6

AV: aortic valve, CABG: coronary artery bypass grafting, DSWI: deep sternal wound infection, MV: mitral valve, TV: tricuspid valve, type 2 A: without involvement of the bone or retrosternal tissue, 2 C: involves bone and retrosternal tissue.

### Microbiology of DSWI

Deep tissue cultures were positive in all 10 patients (100%). The most common pathogens were coagulase-negative staphylococci (7 patients), followed by *Staphylococcus aureus* (4 patients) (both strains of staphylococci were cultured in 1 patient). No methicillin-resistant *Staphylococcus aureus* (MRSA) was isolated. In cases of mixed wound infection, the other pathogen was *Enterococcus faecalis* in one patient, and Gram-negative bacteria (*Serratia marcescens* and *ESBL-producing Klebsiella pneumoniae*) in two patients. These infections required combination antibiotic therapy according to the sensitivity of the etiological agent.

### DSWI outcome data

The mean interval between heart surgery and DSWI diagnosis was 21 ± 17 days (median: 12 days, range: 5–58 days). The mean duration of negative pressure treatment was 10 ± 5 days. The mean number of wound revisions was 4 ± 1 (range: 2–6). Secondary sternal re-osteosynthesis was performed using sternal wires in five patients, and using metal plates in the other five patients. At the time of secondary wound closure, the mean C-reactive protein concentration was 41 ± 26 mg/l. The mean total hospital stay was significantly longer for patients with DSWI (46 ± 11.6 days) compared to patients without infection (14 ± 11.7 days) (*p* < 0.001). There was no in-hospital or 90-day mortality. The predicted mortality according to the EuroSCORE II was 7.7%. Two patients experienced a late recurrence of DSWI that was successfully treated. All-cause mortality was 4.8% during a median follow-up of 2.5 years (range: 0.3–2.9 years). Among a total of 21 DSWI cases (including vancomycin-untreated cases) one patient died from multiorgan failure due to infective endocarditis of the aortic valve.

### Vancomycin pharmacokinetics and wound penetration in NPWT patients

**Figure 1.**
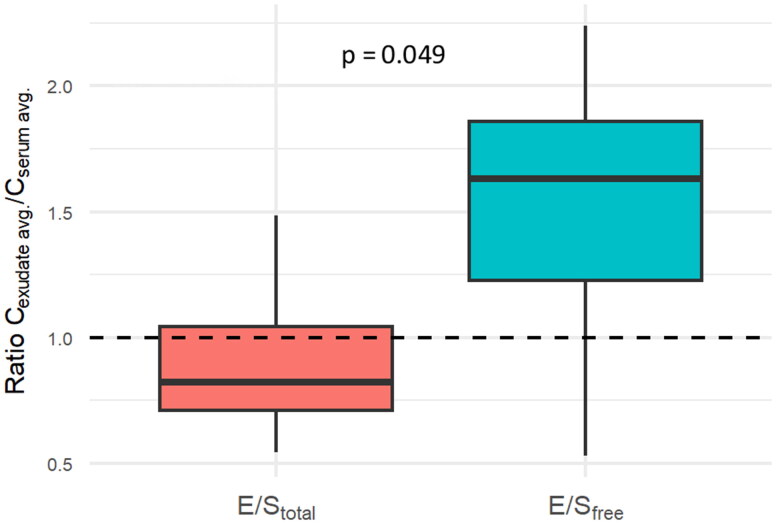
Wound penetration of vancomycin during NPWT on the first sampling day (calculated as the ratio of vancomycin average exudate concentration to average serum concentration). C_exudate avg._: vancomycin exudate average concentration (mg/l). C_serum avg._: vancomycin serum average concentration (mg/l). E/S_total_: ratio of total (bound + unbound) vancomycin average exudate concentration to total (bound + unbound) vancomycin average serum concentration. E/S_free_: ratio of free (unbound) vancomycin average exudate concentration to free (unbound) vancomycin average serum concentration. NPWT: negative pressure wound therapy.

**Figure 2.**
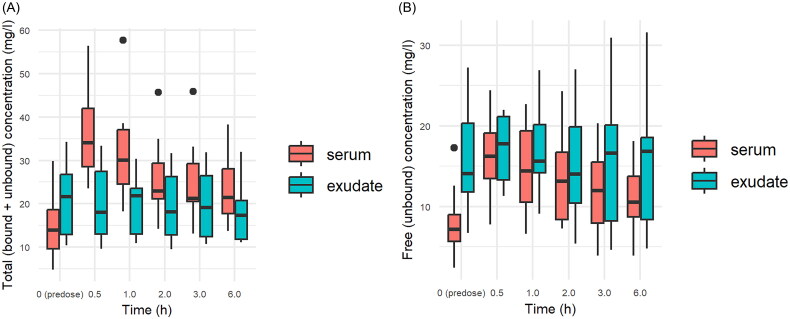
Vancomycin concentration profile in serum and exudate on the first sampling day. **(A)** Total (bound + unbound) concentration. **(B)** Free (unbound) concentration.

**Figure 3.**
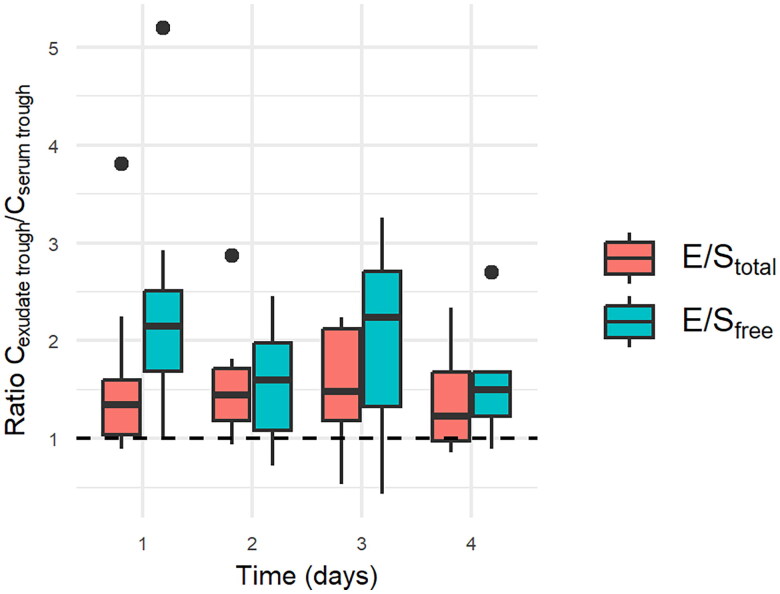
Vancomycin penetration into the wound during NPWT on four consecutive days (including first sampling day, calculated as the ratio of vancomycin trough exudate concentration to trough serum concentration). C_exudate trough_: vancomycin trough exudate concentration (mg/l). C_serum trough_: vancomycin trough serum concentration (mg/l). E/S_total_: ratio of total (bound + unbound) vancomycin trough exudate concentration to total (bound + unbound) vancomycin trough serum concentration. E/S_free_: ratio of free (unbound) vancomycin through exudate concentration to free (unbound) vancomycin through serum concentration. NPWT: negative pressure wound therapy.

Vancomycin was administered for a median of 20 days (range: 8–28 days). On the first sampling day of the study, the total (bound + unbound) average vancomycin concentration was 22.5 ± 8.9 mg/l (range: 10.4–44.3) in serum, compared to 19.5 ± 7.0 mg/l (range: 11.2–30.4) in exudate. The free (unbound) average vancomycin concentration was 10.2 ± 4.4 mg/l (range: 4.2–18.3) in serum, compared to 17.1 ± 6.8 (range: 5.3–28.3) in exudate. The ratio of average wound exudate concentration to average serum concentration was higher for the free (unbound) concentration (1.51 ± 0.53) than for the total (bound + unbound) concentration (0.91 ± 0.29) (*p* = 0.049) (Figure 1). The percentage of free (unbound) vancomycin was higher in wound exudate than in serum (0.79 ± 0.19 vs. 0.46 ± 0.16; *p* = 0.04). In contrast to the time profile of serum vancomycin concentration, the exudate vancomycin concentration showed only minimal fluctuation on the first sampling day (Figure 2). On the following three consecutive sampling days, good vancomycin wound penetration was maintained (measured as the ratio of vancomycin through exudate concentration to vancomycin through serum concentration) (Figure 3).

Three patients experienced vancomycin-induced nephrotoxicity, of whom two developed acute kidney injury (eGFR decreased to 0.32 and 0.57 ml/s/1.73 m^2^) with renal function recovery at the time of hospital discharge. The third patient with vancomycin-induced nephrotoxicity suffered acute-on-chronic kidney injury requiring haemodialysis (at eGFR 0.15 ml/s/1.73 m^2^) and subsequently died (as mentioned above). Predisposing factors associated with vancomycin nephrotoxicity were identified in all three patients. All of them had type 2 diabetes mellitus (complicated by diabetic nephropathy in the third patient). Two were treated with furosemide at doses ranging from 10–750 mg/day. The patients were not concomitantly taking other nephrotoxic drugs. Their serum trough vancomycin concentrations ranged from 20–25 mg/l at the time of acute kidney injury.

## Discussion

DSWI is a serious and feared complication following open-heart surgery. It is associated with prolonged hospital stay, increased cost of care, and significantly increased in-hospital mortality and reduced long-term survival [1–5, 23]. In contrast to previous studies, here we observed no mortality within 90 days after surgery or DSWI-related mortality.

Essential components of DSWI treatment include thorough debridement, NPWT, and culture-directed antibiotics. NPWT accelerates healing of the sternal defect and significantly reduces mortality [3, 24], and is now considered first-line treatment. Notably, NPWT prepares the wound bed for secondary closure by reducing oedema, promoting granulation tissue formation and perfusion, and removing exudate and bacteria [25]. In patients with skin ulcers, skin defects, burns, and traumatic wounds, the migration rate of several antibiotics (including vancomycin) to exudate is reportedly higher in patients treated with NPWT compared to those without NPWT [14, 15]. Skhirtladze et al. investigated patients with soft tissue infection complicating cardiac surgery, who were treated with vancomycin without NPWT [26]. Using the microdialysis method, they found that vancomycin concentrations in the interstitial fluid reached only 10% of the serum concentration in diabetics, and 30% of the serum concentration in non-diabetics.

Few studies have investigated the effects of NPWT on antibiotic wound penetration in patients undergoing various types of surgery [14, 15]. To our knowledge, this is the first study to investigate vancomycin wound penetration in patients receiving NPWT for DSWI after cardiac surgery. We observed that vancomycin penetrated into the wound exudate of patients, with an exudate-to-serum ratio of around or above 1.0. Both the total and unbound exudate-to-serum ratios showed good vancomycin penetration to wound exudate. In a small study of eight patients with skin ulcers or skin defect wounds treated with NPWT, the mean vancomycin concentration in exudate was 67% of the serum vancomycin concentration [14]. In a prospective observational study of burn and trauma patients treated with NPWT, the ratio of vancomycin wound exudate concentration to average plasma concentration was determined to be 1.86 ± 0.70 [15]. Our present results regarding vancomycin penetration are in the range between the findings of these two previous studies.

Notably, in both previous studies, only total vancomycin serum and exudate concentrations were measured and used to calculate the penetration ratio. However, only the free fraction of the antibiotic can cross the cell membrane and interact with the microbe. Vancomycin plasma protein binding reportedly ranges from 10–80% (mean, 55%) [27]. When we calculated the penetration ratio in our study, using the free (unbound) vancomycin concentration yielded a higher vancomycin exudate-to-serum wound penetration ratio. Some previous studies have reported lower protein levels in exudate compared to serum [28], which is in accordance with our finding of a significantly higher vancomycin-free (unbound) fraction in exudate than in serum. Thus, more vancomycin is available to interact with bacteria in wound exudate compared to in serum.

The vancomycin trough exudate and serum concentrations were measured on three consecutive days to evaluate vancomycin penetration into the wound over time. On all study days, good vancomycin wound penetration was maintained, with a vancomycin exudate-to-serum ratio above 1.0 (calculated from vancomycin trough concentrations).

Both vancomycin total and free average concentrations showed high inter-patient variability. The initial dose was determined according to the patient’s body weight, which was consequently adjusted according to TDM rules, to achieve the desired target serum concentration after the first sampling day. Due to many factors influencing vancomycin serum concentrations, the high inter-patient variability of vancomycin serum concentrations is a well-known and common issue that justifies the introduction of TDM into routine clinical practice [29].

The present study has several limitations. It was a single-centre observational study, with a small sample size. Due to the nature of DSWI, only 10 patients were included in the study. Hence, the reported *p*-values, though they are below the standard 0.05 level, should be taken as a statement on the probability of the Type I error and not as a definitive affirmation of significance. Because of the clear superiority of NPWT over the previously used conventional treatment, it was not possible to ethically randomize patients with DSWI to the groups with and without NPWT treatment, at the time of enrolment in the study. Therefore, direct comparison of tissue vancomycin concentrations between the two groups was not feasible. In the absence of a control group, it was also not possible to comment on whether treatment including NPWT was more effective compared to treatment without NPWT. Another bias might be related to the high variability of exudate flow among NPWT patients. In three of ten patients, the scarcity of exudate meant that we could only measure the total concentration, and not the free fraction.

## Conclusion

Our present results confirm that DSWI can be effectively treated with a combination of appropriate surgical, negative pressure, and antibiotic therapies, yielding excellent survival and a low failure rate, despite a significantly longer hospital stay. Vancomycin effectively penetrated into wound exudate in patients receiving NPWT to treat post-sternotomy DSWI after open-heart surgery. NPWT promotes the penetration of vancomycin into the infection site. In this study, vancomycin concentrations in NPWT exudate were higher than previously reported in soft tissue without NPWT, and within the range of concentrations measured in patients treated with NPWT for skin and trauma wounds.

## Data Availability

The data that support the findings of this study are available from the corresponding author, [Martin Kolek], upon reasonable request.
